# The effects of vildagliptin compared with metformin on vascular endothelial function and metabolic parameters: a randomized, controlled trial (Sapporo Athero-Incretin Study 3)

**DOI:** 10.1186/s12933-017-0607-6

**Published:** 2017-10-10

**Authors:** Naoyuki Kitao, Hideaki Miyoshi, Tomoo Furumoto, Kota Ono, Hiroshi Nomoto, Aika Miya, Chiho Yamamoto, Atsushi Inoue, Kenichi Tsuchida, Naoki Manda, Yoshio Kurihara, Shin Aoki, Akinobu Nakamura, Tatsuya Atsumi, SAIS Study Group, Y. Edagawa, M. Oita, A. Kondo, K. Takahashi, H. Sugawara, M. Dan-noura, Y. Takano, N. Kitao, N. Koyanagawa, A. Miya, K. Yamamoto, C. Yamamoto, H. Nomoto, Y. Kameda, KY Cho, A. Nakamura, H. Miyoshi, T. Atsumi, T. Watanabe, K. Takahashi, N. Koyanagawa, K. Kito, C. Yamamoto, M. Endo, J. Takeuchi, S. Nagai, H. Masuda, A. Inoue, Y. Ono, K. Tsuchida, N. Manda, Y. Kurihara, S. Aoki

**Affiliations:** 1 Division of Rheumatology, Endocrinology and Nephrology, Hokkaido University Graduate School of Medicine, Kita-15 Nishi-7, Kita-ku, Sapporo, Hokkaido 060-8638 Japan; 2 Department of Cardiovascular Medicine, NTT East Japan Sapporo Hospital, Sapporo, Japan; 3 Department of Cardiovascular Medicine, Hokkaido University Graduate School of Medicine, Sapporo, Japan; 4 Clinical Research and Medical Innovation Center, Hokkaido University Hospital, Sapporo, Japan; 5 Japan Community Healthcare and Organization Hokkaido Hospital, Sapporo, Japan; 6 Manda Memorial Hospital, Sapporo, Japan; 7 Kurihara Clinic, Sapporo, Japan; 8 Aoki Clinic, Sapporo, Japan

**Keywords:** Type 2 diabetes, Dipeptidyl peptidase-4 inhibitor, Vildagliptin, Vascular endothelial function

## Abstract

**Background:**

Dipeptidyl peptidase-4 (DPP-4) inhibitors may have protective effects in the early stage of atherosclerosis in patients with type 2 diabetes, although similar effects in advanced atherosclerosis were not shown in recent randomized placebo-controlled studies. Therefore, we investigated the efficacy of DPP-4 inhibitor on endothelial function and glycemic metabolism compared with high-dose metformin.

**Methods:**

In this multicenter, open-labeled, prospective, randomized, parallel-group comparison study, patients with type 2 diabetes treated with low-dose metformin (500–750 mg/day) were enrolled and randomly assigned to a vildagliptin, a DPP-4 inhibitor, add-on group (Vilda) or a double dose of metformin group (high Met) for 12 weeks. Flow-mediated dilation (FMD) and serum metabolic markers were assessed before and after treatment. In addition, glycemic control and metabolic parameters were also assessed.

**Results:**

Ninety-seven subjects (aged 58.7 ± 11.0 years; body mass index, 25.9 ± 4.4 kg/m^2^; HbA1c, 7.3 ± 0.5%; FMD, 5.8 ± 2.6%) were enrolled. Eight subjects dropped out by the end of the study. There were no significant differences between the two groups in baseline characteristics. After 12 weeks, HbA1c was significantly improved in the Vilda group compared with the high Met group (− 0.80 ± 0.38% vs. − 0.40 ± 0.47%, respectively; p < 0.01). However, there were no significant differences in FMD (− 0.51 [− 1.08–0.06]% vs. − 0.58 [− 1.20–0.04]%). Although the apolipoprotein B/apolipoprotein A1 ratio was significantly reduced in the Vilda group compared with baseline (0.66–0.62; p < 0.01), the change did not differ significantly between the two groups (− 0.04 vs. 0.00; p = 0.27). Adiponectin levels were significantly increased in the Vilda group compared with the high Met group (0.75 μg/mL vs. 0.01 μg/mL; p < 0.01).

**Conclusions:**

Regardless of glycemic improvement, combination therapy of vildagliptin and metformin did not affect endothelial function but may exert favorable effects on adipokine levels and lipid profile in patients with type 2 diabetes without advanced atherosclerosis.

**Electronic supplementary material:**

The online version of this article (doi:10.1186/s12933-017-0607-6) contains supplementary material, which is available to authorized users.

## Background

The dipeptidyl-peptidase-4 (DPP-4) inhibitors reduce blood glucose levels in a glucose-dependent manner by increasing endogenous glucagon-like peptide-1 (GLP-1), and have been reported to have protective effects on pancreatic beta cells [1, 2]. Unlike some other anti-diabetic medications, they are associated with a low risk of hypoglycemia and a neutral effect on body weight [3, 4]. GLP-1 relaxes the vascular endothelium in a nitric oxide (NO)-dependent fashion [5], and short-term (105 min) native GLP-1 infusion has been reported to improve vascular endothelial function in patients with type 2 diabetes [6]. Vascular endothelial dysfunction is thought to be an early marker of arteriosclerotic progression [7]. Hence, improvement of vascular endothelial dysfunction may inhibit the progression of arteriosclerosis. In patients with diabetes, the frequency of coronary artery disease is 2–4 times than in non-diabetic patients [8], and the risk of developing cerebral infarction is approximately twofold greater [9]. Suppression of arteriosclerosis is therefore considered an important goal of diabetes treatment. Metformin suppresses macroangiopathy and is a low-cost treatment for diabetes, being recommended as the first-line treatment for type 2 diabetes in Europe and the United States [10–12]. However, DPP-4 inhibitors have been shown to be effective in lean type 2 diabetic patients [13], and are therefore frequently prescribed in Asia, including Japan. It remains unclear whether metformin or a DPP-4 inhibitor should be used to optimally manage the initial stage of arteriosclerosis. Although various studies describe the effects of incretin-related drugs on vascular endothelial function, we previously reported that neither sitagliptin nor liraglutide improve flow-mediated dilatation (FMD) in patients with type 2 diabetes [14, 15]. However, several reports describe different effects of DPP-4 inhibitors on FMD. Suppression of 24-h blood glucose fluctuation by vildagliptin correlated with improved oxidative stress and levels of inflammatory marker compared with sitagliptin [16]. Similar results were reported in Asian patients with type 2 diabetes [17, 18]. Based on these findings, we investigated whether addition of vildagliptin or increased metformin could improve vascular endothelial function in patients with type 2 diabetes and poor glycemic control on low-dose metformin.

## Methods

### Study population

Ninety-seven subjects with type 2 diabetes and well-controlled blood pressure and plasma lipids were enrolled from 13 medical service units located in Sapporo City (SAIS Study Group). The inclusion criteria were as follows: outpatients with type 2 diabetes receiving metformin (500–750 mg/day) for more than 4 weeks in addition to diet and exercise therapy, with or without sulfonylureas or glinides; age 20–75 years; and inadequate glucose control (defined as HbA1c level between 7.0 and 8.5%). We excluded patients diagnosed with atherosclerotic diseases (angina, myocardial infarction, cerebral infarction, and peripheral arterial disease), patients currently receiving insulin therapy, pregnant women, and patients with persistent elevated serum transaminase levels or renal dysfunction.

### Protocol

This was a multicenter, open-labeled, prospective, randomized, parallel-group comparison study. During the study period, diet and exercise therapy were controlled as usual, and changes in dose and frequency of all medications other than vildagliptin and metformin were prohibited during the study period. However, sulfonylureas or glinides could be reduced in patients at risk of hypoglycemia. The primary endpoint of the study was the change in FMD between the treatment groups. The sample size was calculated using the assumption that combination therapy of vildagliptin and metformin or high-dose metformin would improve FMD by 1.5% (SD = 1.8%) and 0.5%, respectively, based on previous single-arm studies using sitagliptin or metformin [19–22]. It was determined that 52 patients were needed for each group to detect a significant difference with 80% power and statistical significance of 5%, assuming unequal variance between groups, based on a two-sample *t* test. Secondary endpoints were the changes in metabolic parameters and surrogate markers of beta cell function. After providing informed consent, patients were assigned to a vildagliptin and metformin combination therapy group (Vilda) or a high-dose metformin treatment group (high Met) by Pocock and Simon’s minimization method according to the characteristic factors of age, body mass index (BMI), HbA1c, and FMD using FileMaker Pro 11 Advanced in the allocation institution. In the Vilda group, vildagliptin (100 mg/day) was administered to patients taking metformin (500–750 mg/day). In the high Met group, the pre-study metformin dose was increased to 1000–1500 mg/day. Strict blood glucose control was performed at each respective medical care center for the 12-week study period. The primary endpoint, FMD, was analyzed at Hokkaido University Hospital before and after the trial by the same technician who was blinded to the treatment groups. In addition, assessments of glycemic control and metabolic parameters were conducted. The subject enrollment period was from April 2013 to March 2016, and the last subject completed the study in Octorber 2016.

### Endothelial function

Evaluation of endothelial function was performed using FMD of the brachial artery according to published guidelines [17–19]. Briefly, FMD was performed in the morning after overnight fasting. From the morning of the examination day, participants refrained from medications, smoking, caffeine, and antioxidant vitamins and received only drinking water prior to FMD measurement. Patients were maintained in a supine position for at least 15 min in a quiet, temperature-controlled room (23–26 °C) before the baseline vascular diameter was measured in the brachial artery of the right arm. Five minutes after systolic compression of the right forearm (50 mmHg over the systolic blood pressure), the cuff was deflated and the vascular diameter was continuously measured. FMD was expressed as a percent change from baseline to peak expansion. Augmentation index and reactive hyperemia index (RHI) were measured using an Endo-PAT device (Itamar Medical Ltd., Cesarea, Israel). Centric systolic blood pressure (cSBP) was measured using HEM-9000AI(OMROM, Kyoto, Japan) according to the manufacturer’s instruction.

### Biochemical analysis

Fasting blood samples were placed on ice immediately after collection and centrifuged at 4 °C, and the resulting supernatants were stored at  −20 °C until assayed. low-density lipoprotein (LDL)-cholesterol levels were calculated using the Friedewald formula. Plasma adiponectin, high-sensitivity C-reactive protein (hs-CRP), glucagon, tumor necrosis factor alpha (TNF-α), N terminal prohormone of brain natriuretic peptide (NT-proBNP), and proinsulin were measured by latex agglutination, nephelometry, radioimmunoassay, enzyme-linked immunosorbent assay, electro-chemiluminescence immunoassay, and radioimmunoassay, respectively (SRL, Inc, Tokyo, Japan).

### Evaluation of relevant factors

Evaluation of beta cell function was based on the proinsulin/insulin ratio. Homeostasis model assessment of insulin resistance (HOMA-IR) was calculated from the fasting plasma glucose and insulin levels according to the following formula: (fasting plasma glucose [mg/dl] × insulin concentration [μU/ml])/405. The levels of derivatives of reactive oxygen metabolites (d-ROM), as indicators of the production of reactive oxygen species, and biological anti-oxidant potential (BAP), as an indicator of antioxidant capacity, were measured as described previously [14, 15].

### Statistical analysis

Results are shown as mean ± SD, median (range) or number (%). Differences in baseline characteristics between the two groups were evaluated using Welch’s *t*-test or the Mann–Whitney U test for continuous variables, and the Chi square test for category variables. The Kolmogorov–Smirnov test for normality was used to determine the appropriate statistical test for the continuous variables. The primary endpoint was analyzed based on the intention-to-treat principle. As the primary analysis, the effects of vildagliptin (Vilda) compared with the high Met group on FMD were assessed by analysis of ANOVA. In Addition, analysis of covariance (ANCOVA) was also performed to adjust for baseline FMD. Multiple imputations were used to address missing outcomes (e.g., FMD after 12 weeks). To impute the missing data, we used the predictive mean matching method, including variables potentially related to the fact that FMD was missing and also variables correlated with FMD. The number of imputations was repeated 100 times. Secondary endpoints were descriptively analyzed for mean baseline and post-treatment changes in endothelial and metabolic parameters in both groups in the complete case population. p values < 0.05 were considered statistically significant. Data were analyzed using SAS version 9.4 (SAS Institute Inc., Cary, NC, USA) for the primary analysis and Ekuseru-Toukei 2012 (Social Survey Research Information, Tokyo, Japan) for other analyses.

## Results

### Baseline characteristics

**Fig. 1:**
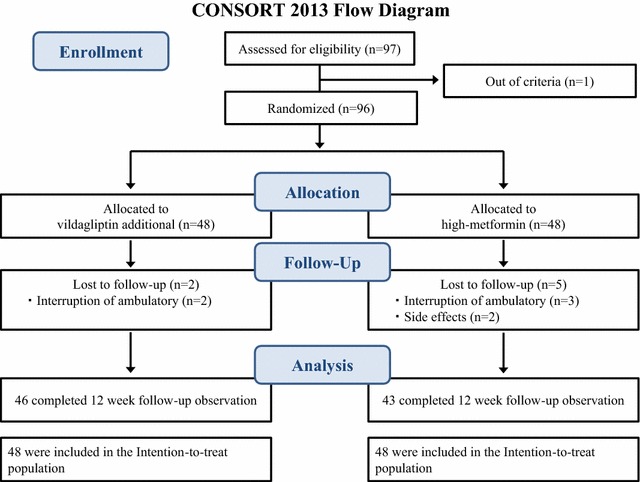
CONSORT flow diagram

A total of 97 subjects were enrolled, but one was subsequently found not to meet the study criteria and withdrew. Of the 96 subjects who underwent the initial examination, none were excluded. Eight subjects dropped out by the end of the study. The reasons for non-completion included interruption of ambulatory visits (n = 6) and side effects of test drugs, such as diarrhea and increased serum levels of creatinine kinase (n = 2) (Fig. 1). The study group included 57 males and 39 females, with an average age of 58.7 ± 11.0 years, BMI of 25.9 ± 4.4 kg/m^2^, and HbA1c level of 7.3 ± 0.5%. At baseline, there were no significant differences between the two groups in age, sex, BMI, blood pressure, biological parameters, prevalence of current smoking, complications of diabetes, and proportion of renin-angiotensin system blockers, statins, and metformin (Table 1).

**Table 1:** Clinical characteristics of the study population

Variables	Vilda	High met	*p* value
	(n = 48)	(n = 48)	
Age (years)	62 (31 to 74)	60 (20 to 74)	0.50*
Male sex (%)	54.2	64.6	0.43
Body mass index (kg/m^2^)	25.7 ± 4.1	26.1 ± 4.7	0.59
Flow mediated dilatation (%)	5.5 ± 2.0	6.1 ± 3.0	0.20
FPG (mM/L)	7.8 ± 1.3	8.1 ± 1.3	0.20
Hemoglobin A1c (%)	7.3 (6.6 to 8.4)	7.2 (6.4 to 8.7)	0.23*
SBP (mmHg)	129.0 ± 13.0	126.6 ± 12.2	0.35
DBP (mmHg)	75.0 ± 9.2	75.5 ± 10.2	0.83
LDL-cholesterol (mg/dL)	112.4 ± 30.0	110.6 ± 28.7	0.76
Current smokers (%)	29.2	27.1	0.90
Hypertension (%)	41.7	43.8	0.89
Dyslipidemia (%)	77.1	68.8	0.43
Angiotensin-converting enzyme inhibitors/angiotensin II receptor blockers (%)	31.3	27.1	0.81
Statin (%)	39.6	35.4	0.80
Diabetic nephropathy (%)	20.8	20.8	1.00
Metformin (mg)	559.5 ± 107.8	595.2 ± 122.9	0.16
Sulfonylureas (%)	18.8	27.1	0.65
Glinides (%)	0	0	1.00

*FPG* fasting blood glucose, *SBP* systolic blood pressure, *DBP* diastolic blood pressure

Values are mean ± SD or median (range). *p* value of Vilda vs. high Met groups

* Mann–Whitney U test was applied to the factors age and HbA1c

### Endothelial function and atherosclerosis

Baseline FMD was 5.48% in the Vilda group and 6.14% in the high Met group. FMD decreased in both groups after treatment, and the changes in FMD were not significantly different between the groups. The primary result did not alter even with ANCOVA adjusted for baseline FMD (p = 0.854) (Table 2). Although no clear relationship was observed between the change in FMD and HbA1c level in either group, the change of apolipoprotein (apo)B/apoA1 ratio decreased significantly in correlation with the change in FMD in the Vilda group (Table 3). Other cardiovascular parameters, such as cSBP, augmentation index, and RHI, remained unchanged (Table 4).

**Table 2:** Comparison of changes in  %FMD before and after treatment in each group

	Vilda (n = 48)	High met (n = 48)	*p* value
Baseline FMD (%)	5.48 ± 2.03	6.14 ± 2.99	
12 weeks FMD (%)	5.14 ± 2.27	5.39 ± 2.29	
ΔFMD (%)	− 0.51 (− 1.08 to 0.06)	− 0.58 (− 1.20 to 0.04)	
Comparison of ΔFMD in ANCOVA	0.08 (− 0.74 to 0.89)	reference	0.854

Values are expressed as mean ± SD or the least square means (95% CI). The least square means were calculated by ANCOVA adjusted for baseline FMD

*FMD* flow-mediated dilation, *ANCOVA* analysis of covariance, *CI* confidence interval

**Table 3:** Relationship between the changes in FMD and other metabolic parameters pre- and post-treatment with vildagliptin or high-dose metformin

Variables	R	*p* value for the Spearman’s rank-correction	R	*p* value for the Spearman’s rank-correction
Medication	Vilda		High met	
BMI	0.038	0.803	0.013	0.932
HbA1c	− 0.131	0.386	− 0.053	0.735
cSBP	− 0.069	0.651	0.050	0.751
Proinsulin/IRI ratio	− 0.060	0.694	− 0.046	0.770
LDL-cholesterol	− 0.249	0.095	0.101	0.521
ApoB/ApoA1 ratio	− 0.326	0.026	− 0.069	0.659
Adiponectin	0.049	0.746	0.124	0.432

*FMD* flow-mediated dilation, *BMI* body mass index, *cSBP* centric systolic blood pressure, *IRI* immunoreactive insulin, *ApoB* apolipoprotein B, *ApoA1* apolipoprotein A1, *BAP* biological antioxidant potential

**Table 4:** Comparison of the effects on other parameters between vildagliptin and high-dose metformin

Variables	Vilda (n = 46)		High met (n = 43)		*p* value
	Baseline	After 12 weeks	Baseline	After 12 weeks	
BMI (kg/m^2^)	25.8 ± 4.1	25.6 ± 4.3	25.8 ± 4.6	25.4 ± 4.6**	0.25
SBP (mmHg)	127.5 ± 11.3	126.4 ± 9.9	127.2 ± 12.5	127.8 ± 11.1	0.51
DBP (mmHg)	74.7 ± 9.1	73.1 ± 8.2	76.0 ± 10.6	75.8 ± 9.4	0.51
Cardiovascular functions					
cSBP (mmHg)^a^	138.2 ± 17.2	134.0 ± 16.5**	136.8 ± 20.3	131.0 ± 16.7**	0.52
Augmentation index (%)	26.5 (− 38.0 to 88.0)	24.0 (− 19.0 to 69.0)	17.0 (− 9.0 to 68.0)	20.0 (− 12.0 to 57.0)	0.83
RHI^a^	1.74 (1.16 to 3.52)	1.77 (1.15 to 4.32)	1.73 (0.86 to 3.16)	1.73 (0.95 to 3.99)	0.37
Biochemical parameters					
FPG (mM/L)	7.8 ± 1.3	6.6 ± 0.9**	8.0 ± 1.2	7.2 ± 1.0**	0.09
Hemoglobin A1c (%)	7.25 (6.60 to 8.40)	6.45 (5.70 to 7.20)**	7.20 (6.40 to 8.70)	6.80 (6.00 to 8.20)**	< 0.01
IRI (µU/mL)	5.8 (1.1 to 17.3)	6.1 (1.3 to 21.5)	5.0 (1.0 to 18.8)	5.1 (1.0 to 16.7)	0.92
Proinsulin/IRI ratio	0.66 (0.19 to 2.12)	0.50 (0.20 to 0.98)**	0.63 (0.24-2.20)	0.56 (0.07-2.04)**	0.19
HOMA-IR^a^	1.88 (0.32 to 5.81)	1.74 (0.33 to 6.00)*	1.80 (0.28 to 9.19)	1.64 (0.22 to 6.72)	0.45
Glucagon (pg/mL)	164 (114 to 337)	155 (112 to 290)	170 (105 to 325)	158 (105 to 275)*	0.73
LDL-cholesterol (mg/dL)	107.9 (55.6 to 185.0)	100.5 (42.2 to 160.4)*	105.8 (53.2 to 180.2)	99.2 (40 to 170.3)*	0.64
HDL-cholesterol (mg/dL)	52.0 (28.0 to 87.0)	49.5 (28.0 to 85.0)	55.0 (35.0 to 105.0)	52.0 (35.0 to 98.0)	0.75
Triglyceride (mg/dL)	102.5 (46.0 to 289.0)	106.0 (48.0 to 241.0)	104.0 (27.0 to 369.0)	102.0 (24.0 to 565.0)	0.38
ApoA1 (mg/L)	139 (95 to 227)	142 (100 to 224)	150 (103 to 224)	146 (112 to 223)	0.14
ApoB (mg/L)	91 (49 to 148)	87 (32 to 131)**	92 (47 to 145)	89 (52 to 146)	0.06
ApoB/ApoA1 ratio	0.66 (0.29 to 1.13)	0.62 (0.21 to 1.29)**	0.60 (0.31 to 1.13)	0.60 (0.30 to 1.15)	0.27
Adiponectin (µg/mL)^a^	6.25 (1.90 to 13.10)	6.80 (1.90 to 15.50)**	6.45 (1.60 to 16.90)	5.90 (1.90 to 16.90)	< 0.01
NT-proBNP (pg/mL)	31 (6 to 294)	26 (6 to 414)	26 (5 to 209)	24 (5 to 169)	0.27
TNF-α (pg/mL)^b^	1.10 (0.70 to 2.10)	1.10 (0.60 to 2.40)	1.25 (0.60 to 1.90)	1.05 (0.60 to 2.10)*	0.15
log hsCRP (ng/mL)	2.88 ± 0.54	2.86 ± 0.54	2.74 ± 0.48	2.63 ± 0.48*	0.19
log albuminuria (mg/g.Cre)	1.04 (0.32 to 2.51)	0.93 (0.41 to 2.67)	0.98 (0.53 to 2.38)	0.85 (0.45 to 2.59)*	0.18
Oxidative stress					
d-ROMs	334.5 (230 to 525)	344.0 (197 to 527)	319 (205 to 488)	318 (143 to 494)	0.69
BAP	2369 (1853 to 3085)	2410 (1806 to 4201)	2447 (1470 to 4622)	2445 (1113 to 3676)	0.30

*BMI* body mass index, *SBP* systolic blood pressure, *DBP* diastolic blood pressure, *cSBP* centric systolic blood pressure, *RHI* reactive hyperemia index, *FPG* fasting blood glucose, *IRI* immunoreactive insulin, *ApoB* apolipoprotein B, *ApoA1* apolipoprotein A1, *NT-proBNP* N terminal prohormone of brain natriuretic peptide, *TNF-α* tumor necrosis factor alpha, *hsCRP* high-sensitivity C-reactive protein, *d-ROMs* reactive oxygen metabolites-derived compounds, *BAP* biological antioxidant potential

Values are mean ± SD or median (range). *p* value mean changes between baseline and the end of the study (Welch’s *t* test or Mann–Whitney U test). * *p* < 0.05 and ** *p* < 0.01 between baseline and the end of the study (paired-sample *t* tests or Wilcoxon signed rank test)

^a^Data from 88 patients (Vilda, N = 45; high-Met, N = 43)

^b^Data from 88 patients (Vilda, N = 46; high-Met, N = 42)

### Glycemic control and metabolic factors

After the treatment period of 3 months, BMI was significantly decreased in the high Met group but there was no significant difference in the change in BMI between the Vilda and the high Met groups. HbA1c significantly improved in the Vilda group compared with the high Met group (p < 0.01). HOMA-IR also significantly improved in the Vilda group (p = 0.01). Serum glucagon level, TNF-α, and hsCRP were significantly reduced in the high Met group (p = 0.04, 0.02, and 0.03, respectively), and the proinsulin/insulin ratio significantly improved in both groups after treatment, although there were no significant differences between the groups. Changes in the oxidative stress parameters, d-ROM and BAP, were not observed in either group. With regard to lipid profile, LDL-cholesterol levels were reduced in both groups even though lipid-lowering agents were sustained without any modification during the study period, but there was no significant difference between the two groups. The apoB/apoA1 ratio was significantly reduced in the Vilda group at 12 weeks compared with baseline (p < 0.01), but did not significantly differ between the two groups (p = 0.27). Interestingly, adiponectin level was significantly increased in the Vilda group compared with the high Met group (0.75 μg/mL vs. 0.01 μg/mL, respectively; p < 0.01) (Table 4).

## Discussion

Several reports have suggested that incretin-based agents may possess protective effects against atherosclerosis [5, 6, 23–25]. However, some randomized clinical trials have reported that DPP-4 inhibitors failed to reduce cardiovascular events compared with placebo in patients with type 2 diabetes [26–28]. The European Diabetes Association and the American Diabetes Association recommend that metformin is prescribed as a first-line drug regardless of body weight, and DPP-4 inhibitors are increasingly being used as second-line treatment as add-on to metformin [29]. In Japan, fewer patients are prescribed metformin and the daily doses are lower than in Europe and the United States, but since high-dose metformin has been covered by insurance, the number of prescriptions and the daily dosage has gradually increased. In the United Kingdom prospective diabetes study (UKPDS) 34, a significant risk reduction was observed for metformin in diabetes-related end points, all deaths, and stroke, as compared with insulin [10]. A sub-analysis of patients with diabetes in the prevention of restenosis with tranilast and its outcome (PRESTO) trial reported a significant risk reduction with metformin compared with insulin or sulphonylureas in major adverse cardiac events, myocardial infarction, and total deaths [30]. In support of these reports, improvements in vascular endothelial function [21], increased GLP-1 concentration [31], and, in mouse studies, increased GLP-1 receptors in islets [32] have been reported for metformin. Taken together, combination therapy of metformin and GLP-1 related agents appear to be clinically effective in enhancing the GLP-1 effect, with a positive influence on vascular endothelial function. However, we observed no improvement in FMD with vildagliptin or metformin over 12 weeks in our study. Although improvement of endothelial dysfunction with a single administration of vildagliptin has been reported [33], in fact the reported short-term effects of treatment with DPP-4 inhibitors on FMD have been controversial [20, 34–38]. Because some studies over a longer period showed that DPP-4 inhibitors have an anti-atherosclerosis effect [39–41], the length of the study period may be a concern. However, one study reported that adjunctive sitagliptin added to conventional antihyperglycemic drugs in patients with type 2 diabetes did not alter endothelial function evaluated by FMD during a 2-year study period [42]. Several explanations can be hypothesized regarding these conflicting results. First, DPP-4 inhibitors are multitarget compounds, and their activity is therefore connected with the inhibition of various substrates, leading to undesirable effects. Moreover, DPP-4 inhibitors may exert differential effects on substrate activity in a diabetic versus a normoglycemic setting, and chronic treatment with gliptins exerts progressive changes in metabolic parameters beyond those detected in single-dose administration studies [23]. Moreover, the results of FMD can be confounded by factors such as patients’ background [43], air temperature [44], and concomitant medications [45–48]. In this study, no significant differences were observed between the groups for any of these confounding factors. Additionally, adjusted change in FMD, which was calculated based on baseline FMD values, yielded similar results in both groups. Regarding glycemic control, HbA1c significantly improved in the Vilda group compared with the high Met group. We sought to adjust the dose of metformin to achieve HbA1c of < 7% and achieved this for the high Met group after treatment, with HbA1c of 6.8%. The hypoglycemic effect of vildagliptin was larger than expected, however, and was associated with a significant improvement in HbA1c. The Vilda group did not exert any additional beneficial effect on vascular endothelium despite a greater effect on HbA1c. Because metformin has beneficial effects on vascular endothelium [37, 49–51], there is a possibility that the effect of improving FMD had already been achieved by pretreatment with metformin. Moreover, because the baseline FMD in this population was similar to that of the healthy group [52], it is also possible that the improvement was difficult to obtain by treatment. Indeed, our result demonstrated a negative correlation between the baseline FMD and ΔFMD (Additional file 1: Figure S1). Systolic blood pressure in each group did not change after treatment for 12 weeks, whereas central blood pressure decreased in both groups. As central blood pressure was related to cardiovascular events rather than peripheral blood pressure [53], arteriosclerosis in both groups was beneficially affected. LDL-cholesterol is a major atherogenic lipoprotein which contributes to the development of coronary heart disease, and currently represents the primary target for lipid-lowering therapy for the prevention and treatment of cardiovascular disease [54, 55]. ApoB reflects the amount of cholesterol, (and, to some degree, triglyceride)-containing particles [56, 57]. ApoA1 is the major apolipoprotein associated with high-density lipoprotein (HDL), and is associated with several anti-atherogenic effects [58]. The apoB/apoA1 ratio is also a marker of risk for future cardiovascular disease [59]. In addition to improving lipid by improving blood glucose, it has been reported that anagliptin inhibits lipogenesis in the liver [60]. Another study showed that linagliptin for 16 weeks improved endothelial function evaluated by FMD and that this effect was mediated, at least in part, by reduction in apoB [61]. In this study, the apoB/apoA1 ratio was significantly improved and negatively correlated with the change in FMD in the Vilda group compared with baseline. Moreover, the addition of vildagliptin to metformin significantly improved serum adiponectin levels compared with high-dose metformin. As there was no clear relationship observed between the changes in HbA1c and adiponectin in both groups, the improvement of adiponectin was thought to be independent of HbA1c. Adiponectin is known to exert multifaceted effects, having anti-inflammatory, anti-arteriosclerotic, and anti-diabetic activities [62, 63]. Furthermore, treatment with sitagliptin or vildagliptin has been shown to increase adiponectin levels [14, 20, 64, 65] through a mechanism which may involve decreased oxidative stress or visceral fat [66]. Markers of oxidative stress were altered in neither group in this study. However, we did not evaluate visceral fat, so we could not confirm the potential involvement of this mechanism. In accord with decreasing ApoB/ApoA1 ratio and increasing adiponectin levels, there is a possibility that vildagliptin-induced improvement of lipid metabolism might exert a beneficial effect on cardiovascular function. Consistent with a basic scientific study demonstrating that adiponectin could have an insulin-sensitizing function [67], HOMA-IR improved in the Vilda group in which adiponectin increased. On the other hand, despite a lesser effect on HbA1c levels in the high-Met group, the high-Met group performed better on pro-inflammatory cytokines. This could be related to greater reduction of body weight in the Met group. It was also reported that metformin inhibited inflammatory cytokines by attenuating nuclear factor κB activation via AMPK activation [68], downregulating chemokine ligand 10 and tissue inhibitor of metalloproteinase 1 gene expression, and increasing vascular endothelial growth factor A [50]. It is possible that short-term combination therapy of vildagliptin and metformin has an inhibitory effect on arteriosclerosis. Recent reports show that alogliptin and sitagliptin did not improve the intima-media thickness over 12 months, but did significantly improve it following a 24-month treatment period [39, 40]. Given that centric systolic blood pressure, HOMA-IR, apoB/apoA1 ratio, and adiponectin improved following 12 weeks of treatment with vildagliptin in this study, FMD may also improve with longer vildagliptin treatment.

A major limitation of this study was the failure to enroll the target number of subjects (97 out of 104). Because of the severity of entry criteria, enrollment was not easy in three years. Future studies should thus involve a larger number of institutes, a longer study period, or a simpler protocol. There is a report that FMD was significantly reduced (worsened) by a DPP-4 inhibitor over 6 weeks [34]. Although there was no significant difference in our study over 12 weeks of treatment, given that 7 enrolled patients did not complete the study, we cannot fully rule out significant worsening of FMD by the DPP-4 inhibitor. Another potential limitation of our study was that medication use was not blinded, although the investigator who performed the FMD was unaware of the patient status and medical history. Moreover, HbA1c reduction was significantly greater in the Vilda group than in the high-Met group. This difference may have influenced some results in this study. This difference may have affected to some results in this study. Finally, the 12-week study duration may have been insufficient to assess the change in FMD or atherosclerosis, as stated in the discussion. To resolve these potential issues, our findings should be validated in a double-blind study, using a larger population over a longer period of time (Additional file 2).

## Conclusions

In this study, combination therapy of vildagliptin and metformin did not further improve vascular endothelial function compared with high-dose metformin over 12 weeks. However, combination therapy improved the apoB/apoA1 ratio and increased serum adiponectin levels, suggesting that long-term combined administration of vildagliptin and metformin may have an inhibitory effect on arteriosclerosis (Additional file 3).

## Additional files

**Additional file 1: Figure S1.** Relationship between the changes in FMD and baseline FMD with vildagliptin or high-dose metformin.

**Additional file 2.** All raw data in this study at week 0.

**Additional file 3.** All raw data in this study at week 12.

## Abbreviations

ANCOVA: analysis of covariance
apoA1: apolipoprotein A1
apoB: apolipoprotein B
BAP: biological antioxidant potential
BMI: body mass index
cSBP: centric systolic blood pressure
DBP: diastolic blood pressure
DPP-4: dipeptidyl-peptidase-4
d-ROMs: reactive oxygen metabolites-derived compounds
FMD: flow-mediated dilation
FPG: fasting blood glucose
GLP-1: glucagon-like peptide-1
HbA1c: hemoglobin A1c
HDL: high-density lipoprotein
HOMA-IR: Homeostasis model assessment of insulin resistance
hsCRP: high-sensitivity C-reactive protein
LDL: low-density lipoprotein
NO: nitric oxide
NT-proBNP: N terminal prohormone of brain natriuretic peptide
PRESTO: prevention of restenosis with tranilast and its outcome
RHI: reactive hyperemia index
SBP: systolic blood pressure
TNF-α: tumor necrosis factor alpha
UKPDS: United Kingdom prospective diabetes study
