# Supplementary material for: The effects of vildagliptin compared with metformin on vascular endothelial function and metabolic parameters: a randomized, controlled trial (Sapporo Athero-Incretin Study 3)
Source: Cardiovasc Diabetol. 2017 Oct 10;16:125. doi: 10.1186/s12933-017-0607-6 (PMC5634845; doi:10.1186/s12933-017-0607-6)
Supplement: Supplementary file 1 — Additional file 1: Figure S1. Relationship between the changes in FMD and baseline FMD with vildagliptin or high-dose metformin. [file 12933_2017_607_MOESM1_ESM.pptx]

## Slide 1
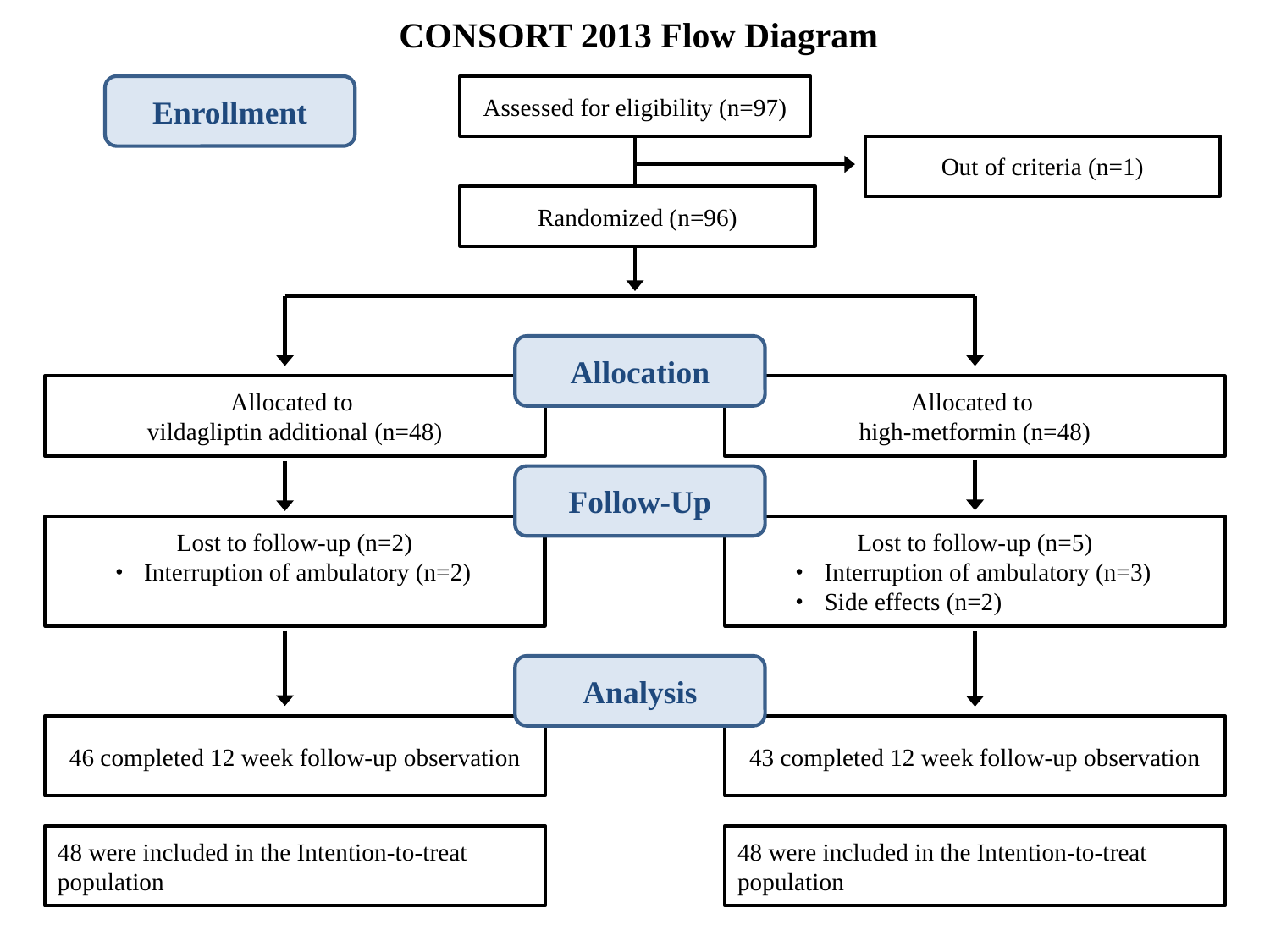

CONSORT 2013 Flow Diagram
Enrollment
Assessed for eligibility (n=97)
Out of criteria (n=1)
Randomized (n=96)
Allocation
Allocated to
vildagliptin additional (n=48)
Allocated to
high-metformin (n=48)
Follow-Up
Lost to follow-up (n=5)
 　 ・ Interruption of ambulatory (n=3)
 　・ Side effects (n=2)
Lost to follow-up (n=2)
 　 ・ Interruption of ambulatory (n=2)
Analysis
46 completed 12 week follow-up observation
43 completed 12 week follow-up observation
48 were included in the Intention-to-treat population
48 were included in the Intention-to-treat population
